# ASSESSING USABILITY OF A MEDICATION ADHERENCE SYSTEM FOR PERSONS WITH MILD COGNITIVE IMPAIRMENT

**DOI:** 10.1093/geroni/igad104.0266

**Published:** 2023-12-21

**Authors:** Renato Ferreira Leitao Azevedo, Timothy Hale, Teresa Warren, Eliza Baby, Jeannie Lee, Kathleen Insel, Wendy Rogers, Raksha Mudar

**Affiliations:** University of Illinois Urbana-Champaign, Urbana, Illinois, United States; University of Illinois Urbana-Champaign (UIUC), Champaign, Illinois, United States; University of Illinois Urbana-Champaign (UIUC), Champaign, Illinois, United States; University of Illinois Urbana-Champaign (UIUC), Champaign, Illinois, United States; The University of Arizona, Tucson, Arizona, United States; The University of Arizona, Tucson, Arizona, United States; University of Illinois Urbana-Champaign, Champaign, Illinois, United States; University of Illinois Urbana-Champaign (UIUC), Champaign, Illinois, United States

## Abstract

Persons with mild cognitive impairment (PwMCI) are at risk of medication nonadherence due to prospective memory deficits. Over time, they are likely to experience accelerated deterioration in cognitive functions and may develop dementia when comorbid treatable health conditions, such as hypertension, are not well managed. A digital therapeutic system called Medication Education, Decision Support, Reminding, and Monitoring (MEDSReM©) has been designed for cognitively normal older adults to support hypertension medication adherence and is currently being tested in a randomized controlled trial. MEDSReM has the potential to support PwMCI with hypertension medication adherence, but it must be optimized specifically for this population. We conducted a cognitive walkthrough to identify usability issues and inform the redesign and optimization of the system for PwMCI. This usability technique is used to evaluate a system from a user’s perspective and is typically conducted by subject matter experts. We had 12 individuals with interdisciplinary expertise in cognitive aging, cognitive impairment, hypertension management, nursing, human factors, and health technology participate in the cognitive walkthrough. Usability issues related to figure-ground contrast, text complexity, and flexibility related to decision making were identified. Overall, recommendations included (1) simplifying the user interface (e.g., color contrast/textual cues), (2) eliminating options related to medication taking actions (e.g., snooze, take later) to mitigate memory-related errors, and (3) providing more direct, prescriptive instructions to minimize decision-making demands. Based on these recommendations, MEDSReM is currently being redesigned for testing with PwMCI. Our approach can inform the design of other mobile health tools.

